# The Impact of Lifetime Work and Non-work Physical Activity on Physical Fitness Among White – and Blue – Collar Retirees: A Cross-Sectional Study

**DOI:** 10.3389/fmed.2021.745929

**Published:** 2021-12-15

**Authors:** Tomasz Trzmiel, Anna Pieczyńska, Ewa Zasadzka, Mariola Pawlaczyk

**Affiliations:** ^1^Department of Occupational Therapy, Poznan University of Medical Sciences, Poznań, Poland; ^2^Department and Division of Practical Cosmetology and Skin Diseases Prophylaxis, Poznan University of Medical Sciences, Poznań, Poland

**Keywords:** occupation, retirement, spirometry, physical activity, handgrip strength (HGS)

## Abstract

**Objective:** The literature offers significant amount of data on the effects of occupational activity on health, with a distinct link between retirement and health among the most frequently tackled topics. Studies on the relationship between past occupational activity and physical fitness among older retirees remain scarce. The aim of the study was to assess the effects of physical activity on physical fitness in white- and blue-collar retirees.

**Methods:** A total of 200 participants (aged ≥60) were included in the study. Lifetime physical activity was assessed using the Lifetime Physical Activity Questionnaire. Mean MET/week/year values of total Physical Activity and for each domain separately (occupational, sports, household) were calculated. Participants were stratified to blue- or white- collar group. Physical performance, hand-grip strength (HGS) and pulmonary function were assessed.

**Results:** Mean total MET/week/year values for the blue- and the white-collar workers were 140.48 ± 55.13 and 100.75 ± 35.98, respectively. No statistically significant differences in physical performance scores were found between the white- and blue- collar groups. Adjustment for age, sex weight and height revealed a statistically significant association between work-related PA FEV*1 in the blue-collar group. White – collar workers presented higher odds ratio for membership in highest quartile in regard to short physical performance battery test score.

**Conclusion:** Only minimal association of type of occupation on physical fitness were found despite statistically significant differences between mean intensity and duration of sports- and work-related lifetime physical activity. These findings may indicate that the type of past work is not an independent factor influencing the state of a person in old age. Large-scale investigations with physically fit and unfit participants, are necessary.

## Background

In the European Union (EU), the number of individuals reporting work-related health problems has been steadily decreasing, from <13% in 2007 to <8% in 2013, for the entire workforce. Regardless, the numbers continue to be high in some EU countries, e.g., Sweden and Finland (20%) (1). The differences stem not only from workload differences for various positions, but also health systems, prevention, and reporting as well as treating work-related health problems (1). An average person devotes up to one-third of their life to work (2, 3). The working environment, physical and emotional strain, or forced positions are only a few of the factors which affect the health of the workers (4–6). The indirect effect of the occupational activity on health, mediated by the impact on the socioeconomic status, has also been reported in the literature (7).

Both, blue- and white-collar jobs can contribute to adverse health outcomes. White-collar workers typically perform sedentary jobs which, due to lack of movement and improper body positioning, may contribute to developing back and neck pain (8). White-collar jobs are frequently associated with markedly elevated stress levels, emotional strain (9), and risk of obesity (10). These factors increase the risk for developing chronic diseases (9). In contrast, blue-collar jobs tend to be more physically demanding and are often correlated with stress, lack of satisfaction, and low socioeconomic status. Blue-collar work has been demonstrated to be a risk factor for developing chronic diseases, heart failure and depression (9). Blue-collar workers are at high-risk for respiratory hazards due to their working conditions and the presence of air pollutants, e.g., dust related to construction or farming jobs (11).

The literature offers an accumulating body of data on the effects of occupational activity on health, with a distinct association between retirement and health among the most frequently tackled topics (12). Physical fitness can be considered as one of the health indicator and is defined, according to The American College of Sports Medicine, as a combination of features and attributes that allow to perform physical activity (13). Studies on the effect of past occupational activity and physical fitness among older retirees remain scarce (14, 15). The analysis of such links continues to present a challenge as health in older age is the sum of a multitude of factors accumulating throughout the course of one's life. Physical activity (PA), both related and unrelated to occupational activity, is an example of one such crucial factor.

According to the World Health Organization (WHO) (16), physical activity (for older people: 150 min./week of moderate-intensity or 75 min./week of vigorous-intensity aerobic) is the key element of “healthy ageing,” as it lowers the risk for developing severe illness and physical disability (17, 18).

PA measuring methods frequently pose a challenge. Self-reported physical activity questionnaires such as IPAQ (International Physical Activity Questionnaire) (19), although uncomplicated, fast and easily applicable, have received a fair share of criticism due to the respondent-related risk of under- or over-estimation. (14, 20) Additionally, they will typically cover the current time or the last few weeks or months before the study. More objective methods such as the use of accelerometers are infinitely more demanding in terms of the cost, not to mention time. Also, the more objective methods do not allow for a retrospective analysis, which is necessary to achieve precise evaluation of the effects of lifetime work-related PA on physical fitness in retirees. Furthermore, it is necessary to take into account other types of PA to avoid selection bias.

Searching for a relationship between the type of work performed in the past and the fitness of retired people may enable the implementation of appropriate activities related to health promotion in groups particularly exposed to a decline in fitness level. The aim of the study was to assess the effects of physical activity on physical fitness in white- and blue-collar retirees.

## Methods

### Participants

**Figure 1:**
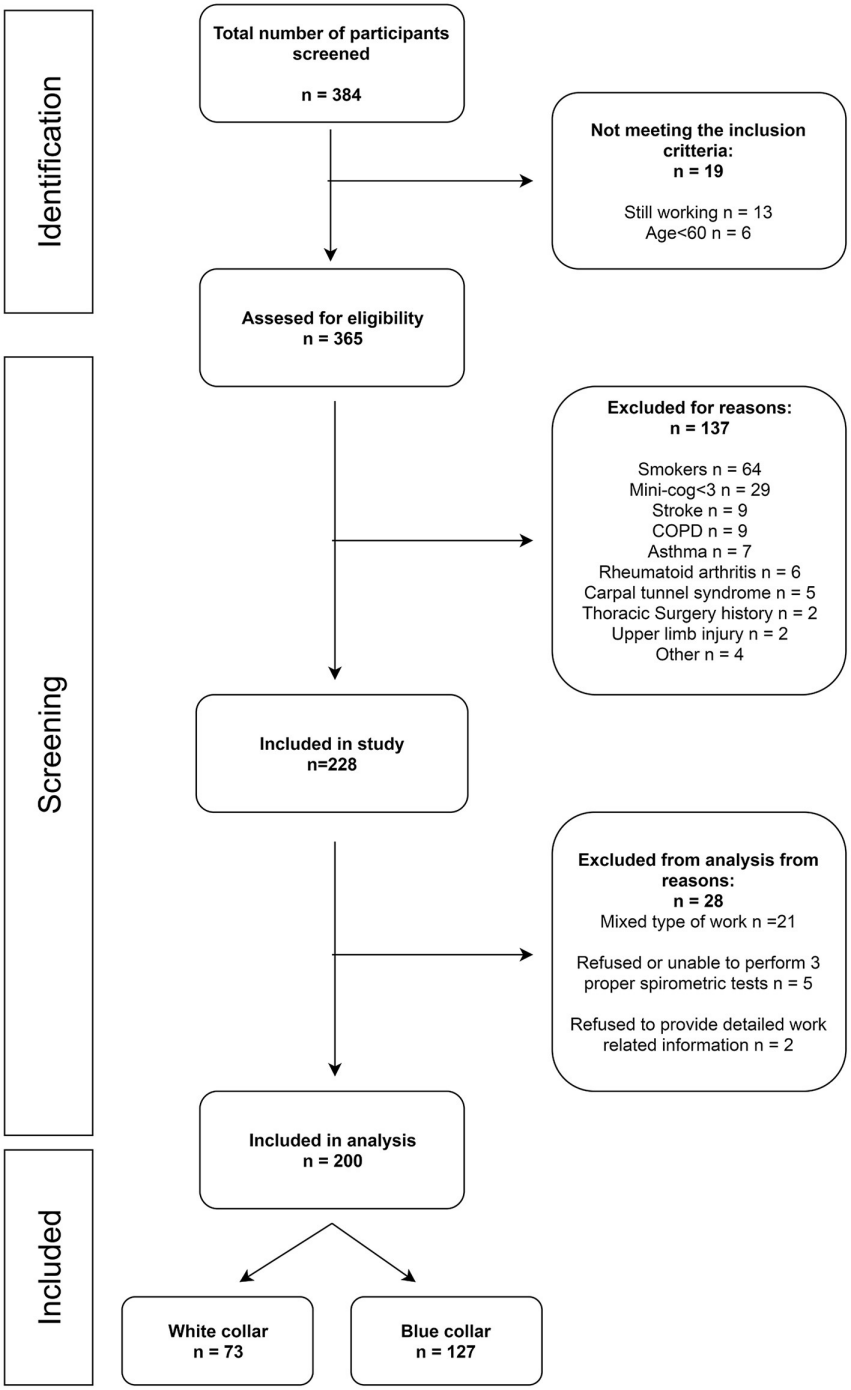
Study participants flow diagram.

The participants were recruited from the general population of the Wielkopolska Region using radio, Internet, and print (leaflets/flyers) media and in cooperation with various organizations for seniors. A total of 200 participants (out of 384 who volunteered to participate) were included in the study (Figure 1). The inclusion criteria were as follows: age ≥60 years and cessation of the occupational activity. The volunteers were interviewed to identify possible exclusion criteria such as diseases and injuries which might negatively affect the upper limb performance and/or respiratory tract function (e. g., stroke, asthma, Chronic Obstructive Pulmonary disease (COPD), history of thoracic surgery, upper limb fracture, brachial plexus injury, brachialgia, history of pulmonary surgery, smoking and impaired cognitive function).

Cognitive functioning was assessed with the Mini-Cog test (21). Mini-Cog consists of two components: short-term recall of three words to test short-term memory and the clock drawing test, with 5 points as the maximum score. The score of <3 points may be indicative of cognitive impairment, so it constituted one of the exclusion criteria in the present study. Permission to use the Mini-Cog test was obtained.

The study was conducted between March 2019 and October 2019. The requirements of the Declaration of Helsinki were followed.

### Measurements

Lifetime physical activity was assessed using the Lifetime Physical Activity Questionnaire (LTPAQ) (22). Lifetime physical activity in the present study was defined as average PA calculated from lifetime physical activity patterns reported in LTPAQ.

The questionnaire consists of 4 domains which measure occupational activity (i.e., work), sports, household and transportation-related physical activity. In the present study, the transportation domain was not analyzed separately but some information collected from that domain were included in the presentation of Sports data. Before the study, all participants received a recall calendar, which facilitated their recall of various kinds of lifetime physical activity, according to the guidelines specified in the LTPAQ COMPREHENSIVE USERS' GUIDE (23).

All types of occupational activity or volunteering performed for the minimum of 8 hours/week, for 4 months (min. 128 hours/year or 2.5 hours/week for a year) were treated as occupational activities. Each respondent submitted data on the age at which s/he started and finished working at each position, including the number of months/year, days/week, hours/day. Next, the intensity of physical exertion was described on a scale from 1 to 4 (Table 1). If the intensity of different tasks varied significantly, the total working time was divided and different intensities were assigned to individual tasks, as per respondent instruction.

**Table 1:** LTPAQ—task intensity.

**Task intensity**	
Intensity category	Work intensity—description
1	Only sitting, with minimal walking.
2	Minimal amount of physical effort (standing and slow walking). No increase in heart rate and no perspiration.
3	Carrying light loads and continuous walking. Heart rate slightly increased, light perspiration.
4	Carrying heavy loads, brisk walking, and climbing. Heart rate substantially increased, heavy sweating.
Intensity category	Household intensity—description
2	Minimal physical effort such as accompanying those activities which are done standing or with slow walking.
3	Not exhausting, slightly increased heart rate, some light perspiration.
4	Increased heart rate, heavy sweating (lifting, moving heavy objects, rubbing vigorously for fairly long periods).
Intensity category	Exercise and sport intensity—description
2	Minimal physical effort
3	Not exhausting, slightly increased heart rate, some light perspiration.
4	Heart rate increased and heavy sweating.

Household Activities included housework, yard work, home repair and childcare, which took the minimum of 7 hours/week for 4 months (or 112 hours total/year or 2.15 hours/week per year). Sedentary household activities such as sewing were not registered (23). The level of intensity of physical exertion for Household Activities was described on a scale from 2 to 4 (Table 1).

As far as Exercise and Sports Activities were concerned, sports activities which were performed for at least 32 hours total/year (or 40 minutes/week per year, or 2 hours/week for 4 months) were included in that category. Next, the Metabolic Equivalent of Task (MET) was assigned to each activity, in accordance with the 2011 Physical Activity Compendium. (24) If a respondent walked/rollerbladed/biked/ran to work, that type of physical activity was also included in the Exercise and Sports Activities.

In order to determine the Total PA MET value, the combined number of the hours spent performing a particular activity was calculated using the following an original formula created for the purpose of this study, based on the LIFETIME TOTAL PHYSICAL ACTIVITY QUESTIONNAIRE COMPREHENSIVE USERS' GUIDE (23) and other publications: (25, 26).


$$\begin{array}{l} YearsxMonthsxDaysxHoursx\;4=TotalLiftimeActivityTime \end{array}$$

The obtained number of the hours was divided by the number of the years of life and the result was again divided by 48 to determine the duration of lifetime activity/week. That result was then multiplied by the MET value of a given task to achieve the lifetime MET/week/year value for that task. The following formula was applied:


$$\begin{array}{l} \frac{\left(TotalLifetimeActivityTime/YearsOfLife\right)}{48}\;*\;MET=MET/week/year \end{array}$$

The obtained values for specific activities from the different domains were summed up to determine the following: Total Lifetime (which means average value of PA based on summed lifetime Occupational, Household and Sports MET/week/year, and the combined Total MET/week/year. Total Lifetime Physical Activity was average value of PA Similarly, after summing up the hours of lifetime physical activity, we received the Lifetime Total h/week/year value - combined and separate for each category.

The International Standardized Classification of Occupations (ISCO-08) (27) was used to stratify the participants into the white- or blue-collar groups, depending on the type of occupational activity reported during the LTPAQ interview. Similar to the methodology proposed by Fukushima et al. (27) the blue-collar group included participants performing physical tasks who had worked as mechanics, farmers, builders, production workers and electricians, whereas the white-collar group comprised office workers, accountants, physicians, managers, engineers, and academics. Participants who could not be classified into the white- or blue- collar group due to the mixed nature of their past work were removed from the analysis.

There is no consensus over physical performance definition, however for the purpose of this study definition proposed by other authors was adopted. According to Beaudart et al. (28) physical performance can be defined as “an objectively measured whole body function related with mobility” which involved not only muscle function measures but also other organs and systems (including cardiorespiratory system). Therefore, in the present study hand-grip strength (HGS), spirometry and Short Physical Performance Battery test (SPPB) results constituted the indicators of the physical performance.

Physical performance, was assessed using the SPPB (29). The test consists of three tasks: rising from a chair and sitting down with five repetitions, a hierarchical test of balance, and a short walk at the usual pace. Each SPPB component is scored from 0 to 4, with 0 representing the inability to perform the test and 4 representing the highest category of performance. The highest possible score is 12, indicating the best body function (29, 30).

HGS of the dominant hand was tested using the JAMAR hydraulic dynamometer. The measurements were performed in accordance with the recommendations of the American Society of Hand Therapists (ASHT) (31). During the measurements, the subject was sitting in a chair without a backrest and armrests, with feet resting on the floor, parallel to each other, hip and knee joints set at right angles, arms adducted and touching the torso, elbow joint bent to 90°, forearm in a neutral position, wrist straightened in the range of 0° to 30°. The subject performed a maximum handgrip and held it for 6 s. The dominant hand was tested three times, with a 1-min break between each measurement. The best result was used for further analysis.

Pulmonary function was assessed using spirometry. Spirometry was performed using non-turbine Piston PDD 301 spirometer, in accordance with the American Thoracic Society and European Respiratory Society recommendations (32). Spirometer software indicated whether the measurement was correct and the test was valid, or if repeat test was required. Single-use disposable filter mouthpieces were used. FVC (Forced Vital Capacity), FEV*1 (Forced Expiratory Volume in the 1st second) and IVC (Inspiratory Vital Capacity) parameters were tested. During the tests, the participants were vigorously encouraged by the technician to give it their best effort while inhaling and exhaling. The test was repeated up to 8 times (with 1-min break between the manoeuvers) until three correct measurements were achieved. Both, the technician who performed the test and the software indicated whether the measurement was correct, and the test was valid, or if repeat test was required. Mean score was used for further analysis. This study is part of a project “Type of work performed in the past and physical fitness at retirement.”

### Statistical Analysis

The statistical analysis was conducted using Statistica 13 (StatSoft, Polska). Mean, median and standard deviation were used to describe group characteristics. In the absence of normal distribution of the investigated variables, U-Mann-Whitney test was used to compare the white- and blue-collar groups. HGS and spirometry results were adjusted for age, sex, weight and height. In order to investigate the effects of PA on HGS and spirometry results for every variable, statistical analysis was conducted using the General Linear Regression (GLR): GLR - without control bias, Model 1 –controlled for age and sex, and Model 2 - controlled for age, sex, weight and height. The minimal study sample of 96 persons was calculated with assumption of z = 1.96 and error = 10%. The *p*-value of <0.05 was considered as statistically significant.

## Results

A total of 200 people (127 blue- and 73 white – collar retirees) were included in the study. Out of them, 107 were female. Mean patient age was 70.11 ± 6.55 years (69.93 ± 6.49 and 71.36 ± 6.51 for the blue- and white-collar groups, respectively). No statistically significant differences were found between blue- and white-collar workers as far as retirement duration was concerned (p=0.18, 10.49 ± 8.73 and 12.12 ± 9.54, respectively). Mean MET/week/year values for the blue- and the white-collar workers were 140.48 ± 55.13 and 100.75 ± 35.98, respectively. The MET/week/year values for Work were 96.54 ± 53.11 and 52.57 ± 34.98, respectively. The values and comparison of the mean values for both groups are presented in Table 2. Statistically significant differences were found for age, MET/week/year for Work and Sports, and Total as well as h/week/year for Work and Sports. The odds ratio for the membership in the 1st and 4th quartile of measured variables in association with the type of occupation were presented in Table 3. There were no statistically significant associations between type of occupation and measured variables except SPPB.

**Table 2:** Group comparison and characteristics.

**Variable**	**Blue collar group *n* = 127**	**White collar group *n* = 73**	***p***
	Mean^*^/Median^**+**^ (SD^*^/ IQR^**+**^**)**	Mean^*^/Median^**+**^ (SD^*^ **/ IQR**^**+**^**)**	
Age^*^	69.39 (6.49)	71.36 (6.51)	**0.016**
Weight^*^	79.13 (10.53)	78.71 (16.33)	0.390
Height^*^	168.15 (7.77)	166.22 (8.96)	0.112
IVC^*^	3.08 (0.89)	2.94 (0.79)	0.246
FEV^*^	3.18 (0.92)	4.36 (11.50)	0.330
FEV^*^1^*^	2.36 (0.74)	3.58 (11.82)	0.193
HGS^*^	29.97 (10.50)	27.73 (10.48)	0.099
SPPB^+^	11.00 (3)	12 (2)	0.177
SPPB S^+^	4 (2)	4 (1)	0.265
SPPB B^+^	4 (0)	4 (0)	0.590
SPPB W^+^	4 (1)	4 (0)	0.546
Work – related PA^*^ (MET/week/year)	96.54 (53.11)	52.57 (34.98)	**<0.001**
Household – related PA^*^ (MET/week/year)	36.77 (32.19)	38.48 (27.62)	0.323
Sport – related PA^*^ (MET/week/year)	7.17 (9.71)	9.70 (8.96)	**0.035**
Total Lifetime PA^*^ (MET/week/year)	140.48 (55.13)	100.75 (35.98)	**<0.001**
Work Time^*^ (h/week/year)	27.15 (9.63)	23.04 (8.69)	**0.005**
Household Time^*^ (h/week/year)	10.86 (9.86)	11.49 (8.53)	0.288
Sport Time^*^ (h/week/year)	1.58 (1.69)	2.18 (1.95)	**0.046**
Total Activity Time^*^ (h/week/year)	39.60 (12.10)	36.71 (9.49)	0.163

* Mean (SD), ^+^ Median (IQR), SD, standard deviation; IQR, interquartile range; HGS, Hand Grip Strength(in kG); SPPB/ SPPB S/SPPB B/ SPPB W/, Short Physical Performance Battery total score/SPPB standing up from chair score/ SPPB balance score/ SPPB walking score; IVC, Inspiratory Vital Capacity (in l); FEV, Forced Expiratory Volume (in l); FEV*1, Forced Expiratory Volume in 1st second (in l). Age was expressed in years, Weight in kg and Height in cm. Bold values are statistically significant value (p < 0.05).

**Table 3:** The odds ratio for the membership in the 1st and 4th quartile of measured variables in association with the type of occupation.

	**1st quartile odds ratio OR (95% CI)**		
	**Blue - collar (reference)**	**White** - **collar**	***p***
HGS	1	1.63 (0.75–3.58)	0.221
SPPB	1	**0.36 (0.16**–**0.79)**	**0.012**
IVC	1	0.82 (0.39–1.20)	0.587
FEV	1	0.99 (0.45–2.14)	0.973
FEV^*^1	1	1.39 (0.68–2.87)	0.370
		**4th quartile odds ratio OR (95% CI)**	
HGS	1	1.43 (0.59–3.43)	0.426
SPPB	1	**2.01 (1.07**–**3.75)**	**0.029**
IVC	1	1.02 (0.43–2.42)	0.957
FEV	1	1.36 (0.58–3.19)	0.475
FEV^*^1	1	1.30 (0.57–2.94)	0.538

* Adjusted for age and sex, HGS, Hand Grip Strength; SPPB, Short Physical Performance Battery score; IVC, Inspiratory Vital Capacity; FEV, Forced Expiratory Volume; FEV*1, Forced Expiratory Volume in 1st second. Bold values are statistically significant value (p < 0.05).

Table 4 presents the effects of PA on the investigated parameters. Before adjustments, statistically significant associations were found between Total MET/week/year and FEV and FEV*1. Model 1 also revealed statistical significance between these parameters, whereas Model 2 only between MET/week/year and FEV*1. In the white-collar group, statistically significant associations between PA and SPPB values were found both, before and after adjustments.

**Table 4:** Relationships between total and work lifetime physical activity (in MET/week/year) and the investigated variables.

	**Blue - collar**					**White – collar**				
	**HGS**	**SPPB**	**IVC**	**FEV**	FEV*1	**HGS**	**SPPB**	**IVC**	**FEV**	FEV*1
**Total Lifetime Physical Activity**										
Unadjusted										
β	0.008	−0.012	0.113	**0.232**	**0.269**	0.040	**0.323**	0.059	0.142	−0.001
SE	0.089	0.089	0.089	**0.087**	**0.086**	0.119	**0.112**	0.118	0.118	0.119
CI 95%	−0.169 0.185	−0.189 0.165	−0.063 0.289	**0.059 0.404**	**0.001 0.006**	−0.196 0.277	**0.099 0.547**	−0.177 0.296	−0.092 0.376	−0.238 0.236
p	0.927	0.897	0.206	**0.009**	**0.002**	0.735	**0.005**	0.617	0.231	0.994
Model 1										
β	0.010	−0.084	0.056	**0.169**	**0.214**	0.045	**0.203**	0.042	0.132	0.071
SE	0.066	0.086	0.077	**0.071**	**0.068**	0.069	**0.095**	0.082	0.122	0.115
CI 95%	−0.121 0.141	−0.248 0.080	−0.097 0.209	**0.029 0.310**	**0.080 0.348**	−0.093 0.183	**0.013 0.393**	−0.121 0.205	−0.111 0.375	−0.159 0.302
p	0.882	0.311	0.470	**0.018**	**0.002**	0.520	**0.037**	0.610	0.282	0.539
Model 2										
β	−0.053	−0.115	0.019	0,114	**0.163**	0.046	**0.202**	0.058	0.130	0.066
SE	0.065	0.085	0.079	0.068	**0.067**	0.070	**0.097**	0.071	0.122	0.117
CI 95%	−0.181 0.075	−0.283 0.054	−0.138 0.175	−0.020 0.248	**0.031 0.296**	−0.094 0.185	**0.009 0.395**	0.004 0.058	−0.112 0.373	−0.167 0.299
p	0.415	0.179	0.815	0.094	**0.016**	0.515	**0.041**	0.410	0.288	0.574
**Work-Related Lifetime Physical Activity**										
Unadjusted										
β	**0.303**	0.053	**0.242**	**0.392**	**0.446**	**0.369**	0.112	**0.276**	0.032	−0.034
SE	**0.085**	0.089	**0.087**	**0.082**	**0.080**	**0.110**	0.118	**0.114**	0.119	0.119
CI 95%	**0.134 0.472**	−0.123; 0.230	**0.070 0.413**	**0.229 0.554**	**0.288 0.604**	**0.149 0.589**	−0.123 0.348	**0.049 0.503**	−0.204 0.269	−0.271 0.203
p	**0.001**	0.551	**0.006**	**<0.001**	**<0.001**	**0.001**	0.344	**0.018**	0.785	0.776
Model 1										
β	0.601	−0.069	0.033	**0.177**	**0.222**	0.011	0.094	−0.070	0.045	0.060
SE	0.071	0.090	0.083	**0.077**	**0.073**	0.076	0.107	0.089	0.134	0.126
CI 95%	−0.080 0.202	−0.246 0.108	−0.132 0.198	**0.026 0.329**	**0.078 0.368**	−0.140 0.162	−0.119 0.307	−0.247 0.107	−0.222 0.312	−0.191 0.311
p	0.396	0.442	0.695	**0.022**	**0.003**	0.885	0.382	0.434	0.736	0.636
Model 2										
β	−0.009	−0.107	−0.013	0.103	**0.159**	0.021	0.104	−0.031	0.150	0.050
SE	0.070	0.092	0.086	0.074	**0.073**	0.077	0.109	0.078	0.135	0.128
CI 95%	−0.148 0.130	−0.290 0.075	−0.182 0.156	−0.041 0.250	**0.015 0.303**	−0.133 0.175	−0.114 0.321	−0.187 0.126	−0.254 0.284	−0.206 0.307
p	0.895	0.246	0.883	0.159	**0.031**	0.789	0.344	0.697	0.912	0.696

HGS, Hand Grip Strength; SPPB, Short Physical Performance Battery; IVC, Inspiratory Vital Capacity; FEV, Forced Expiratory Volume; FEV*1, Forced Expiratory Volume in 1st second. Bold values are statistically significant value (p < 0.05).

Work-related physical activity proved to be correlated with HGS, IVC, FEV, and FEV*1 in the blue-collar group and with HGS and IVC in the white-collar group. Model 1 revealed a statistically significant association between work-related PA and FEV as well as FEV*1, while Model 2 only in case of FEV*1 in the blue-collar group. In the white-collar group, neither Model 1 nor Model 2 found a statistically significant association (Table 4).

Total PA time demonstrated an association only with FEV and FEV*1 in the blue-collar group in Model 1 and 2 (Table 5). The number of lifetime work-related hours of PA showed a statistically significant association with all investigated parameters in the blue-collar group before the adjustments. After the adjustments, the effect was detected only for FEV and FEV*1 both, in Model 1 (age and sex) and Model 2 (Model 1 + height and weight). An association between the number of Total work-related PA hours with IVC pre-adjustment and HGS pre-adjustments as well as in Model 1 and 2 was found in the white-collar group (Table 5).

**Table 5:** Relationships between total and work lifetime physical activity (in hours/week/year) and the investigated variables.

	**Blue - collar**					**White - collar**				
	**HGS**	**SPPB**	**IVC**	**FEV**	FEV*1	**HGS**	**SPPB**	**IVC**	**FEV**	FEV*1
**Total Lifetime Physical Activity**										
Unadjusted										
β	−0.138	0.069	0.075	0.097	0.098	−0.136	0.122	0.070	0.189	0.076
SE	0.089	0.089	0.089	0.089	0.089	0.118	0.118	0.118	0.117	0.118
CI 95%	0.314 0.037	−0.107 0.246	−0.101 0.252	−0.080 0.273	−0.078 0.274	−0.370 0.099	−0.113 0.356	−0.166 0.306	−0.044 0.421	−0.160 0.312
p	0.121	0.438	0.400	0.280	0.274	0.253	0.306	0.555	0.110	0.523
Model 1										
β	0.022	0.057	0.146	**0.178**	**0.198**	−0.089	0.039	0.103	0.181	0.115
SE	0.067	0.084	0.078	**0.072**	**0.070**	0.068	0.097	0.080	0.119	0.113
CI 95%	−0.111 0.155	−0.110 0.223	−0.007 0.230	**0.035 0.320**	**0.061 0.335**	−0.224 0.046	−0.155 0.232	−0.057 0.262	−0.057 0.419	−0.111 0.341
p	0.742	0.502	0.061	**0.015**	**0.005**	0.193	0.691	0.203	0.133	0.314
Model 2										
β	−0.024	0.046	0.123	**0.148**	**0.163**	−0.089	0.035	0.136	0.181	0.106
SE	0.065	0.086	0.079	**0.068**	**0.068**	0.069	0.099	0.069	0.120	0.115
CI 95%	−0.153 0.106	−0.124 0.217	−0.034 0.279	**0.013 0.282**	**0.029 0.296**	−0.226 0.049	−0.163 0.232	−0.002 0.274	−0.059 0.420	−0.125 0.336
p	0.715	0.594	0.123	**0.032**	**0.017**	0.201	0.727	0.053	0.137	0.363
**Work-Related Lifetime Physical Activity**										
Unadjusted										
β	**0.329**	**0.205**	**0.319**	**0.397**	**0.418**	**0.290**	−0.125	**0,400**	0.054	0.050
SE	**0.084**	**0.088**	**0.085**	**0.082**	**0.081**	**0.114**	0.118	**0.109**	0.119	0.119
CI 95%	**0.162 0.496**	**0.031 0.378**	**0.151 0.486**	**0.234 0.559**	**0.254 0.577**	**0.064 0.517**	−0.360 0.110	**0.183 0.617**	−0.182 0.291	−0.187 0.286
p	**<0.001**	**0.021**	**<0.001**	**<0.001**	**<0.001**	**0.013**	0.292	**<0.001**	0.648	0.676
Model 1										
β	0.099	0.147	0.158	**0.219**	**0.219**	**−0.179**	−0.142	0.029	0,090	0.151
SE	0.070	0.087	0.081	**0.074**	**0.072**	**0.076**	0.110	0.093	0.139	0.130
CI 95%	−0.039 0.237	−0.025 0.319	−0.002 0.318	**0.072 0.366**	**0.077 0.362**	**−0.330** **−0.028**	−0.362 0.079	−0.156 0.214	−0.187 0.368	−0.108 0.411
p	0.158	0.094	0.053	**0.004**	**0.003**	**0.021**	0.203	0.756	0.519	0.249
Model 2										
β	0.040	0.128	0.126	**0.160**	**0.164**	**−0.172**	−0.139	0.104	0.042	0.138
SE	0.068	0.089	0.083	**0.071**	**0.071**	**0.079**	0.114	0.082	0.142	0.134
CI 95%	−0.095 0.176	−0.049 0.305	−0.038 0.290	**0.020 0.301**	**0.024 0.304**	**−0.329** **−0.016**	−0.367 0.090	−0.059 0.267	−0.237 0.330	−0.131 0.406
p	0.557	0.156	0.132	**0.025**	**0.022**	**0.032**	0.230	0.207	0.745	0.310

HGS, Hand Grip Strength; SPPB, Short Physical Performance Battery; IVC, Inspiratory Vital Capacity; FEV, Forced Expiratory Volume; FEV*1, Forced Expiratory Volume in 1st second. Bold values are statistically significant value (p < 0.05).

## Discussion

In the present study, we evaluated the effects of work-related and work-unrelated physical activity on physical performance among retirees, who were either white-collar or blue-collar workers during their professional activity years. HGS, spirometry and SPPB results constituted the indicators of the physical performance. No statistically significant differences in physical performance were found between the white- and blue-collar groups. The results of this cross-sectional study demonstrated an association between lifetime work-related and work-unrelated physical activity and FEV*1 among blue-collar retired workers. The relationship between work-related PA was detected both, before and after adjusting for age, sex, height and weight. Notably, even though the correlation after the adjustments was weak (β < 0.2), a similar correlation was not observed in the white-collar group.

Hunter et al., (33) demonstrated a positive role of high-intensity physical activity in muscle function. Taylor et al. (34) also reported high-intensity physical activity to be more beneficial for health than low-intensity PA. That association may explain why in our study higher physical activity (expressed in MET as well as in hours) seemed to be a factor which improved the FEV*1 score in the blue-collar group.

Rawashdeh and Alnawaiseh (35), reported a statistically significantly higher FEV*1 in their study group of 72 males undergoing a three-week long high-intensity aerobic training. These authors demonstrated that physical activity improved the airflow in the respiratory tract, which they attributed to the fact that during the training a larger volume of air was introduced into the airways, resulting in a widening of the respiratory tract. Their findings might help and explain our results. In our study, in the blue-collar group, higher work-related MET/week/year resulted in higher FEV*1, which indicates higher-intensity PA. Similarly, higher number of work-related h/week/year, i.e., more hours spent at work, contributed to higher FEV*1. Bertoni et al. (15) showed that blue-collar workers have a tendency to limit their non-work related PA and are more reluctant to engage in sports activities as compared to the white- collar workers, which is consistent with our observations. Statistically significant association between type of occupation and membership in SPPB score quartiles shows, that being retired white – collar worker doubles the odds for membership in highest quartile regarding SPPB score and lowering odds for membership in lowest quartile (OR = 0.36). However, the clinical importance of association presented in studied group could be reduced because participants SPPB scores were relatively high and most participants were considered as independent (SPPB score ≥ 10). For this regard more studies, involving less independent participants should be conducted in future. The difference between blue – and white collar group can also be explained in the ground of abovementioned patterns of participation in sport activities (15).

In the white-collar group, total exertion score (MET week/year) correlated with the SPPB results, indicating that higher PA corresponded to higher SPPB scores, both before and after the adjustments (Model 1 and 2).In the blue-collar group, statistically significant relationships were found between all of the investigated parameters and the number of hours spent at work before the adjustments. However, the relationship was no longer statistically significant after adjusting for age and sex. The effect of age and sex on the physical function decline as well as the negative impact of age on lung function have been described by different authors (36, 37). Navarro et al. (38) reported that lung function declines 1% per year after the age of 25 years. Various mechanisms of lung function decline have been proposed, especially disturbed respiratory mechanics caused by altered thoracic shape resulting from a progressing spinal kyphosis, impaired diaphragm function, restricted chest and pulmonary alveoli expansion due to loss of elasticity (39, 40).

Importantly, a statistically significant relationship between the number of hours spent at work and HGS in the white-collar group became negative after the adjustments, indicating that longer time spent at work might have a detrimental effect on the health of white-collar workers. Saidj et al. (41) in their study of 2,544 working adults (aged 18–69), evaluated the relationship between sitting position at work and during leisure time and found a negative correlation between these variables and HGS in subjects aged <50 years. However, no such relationship between sitting time at work and HGS was observed, which led them to the conclusion that sedentary behaviors during leisure time are more harmful than at work. The differences between their results and our findings might stem from the fact that we did not evaluate sitting time but only MET or the number of hours dedicated to different types of work.

Duong et al. (42) in their study conducted in a group of 126 359 adults from 17 countries demonstrated a relationship between mortality and FEV*1 decline. Impaired FEV*1 contributed to one-fourth of the deaths and one-sixth of cardiovascular incidents. According to these authors, impaired FEV*1 contributed to the cardiovascular incidents significantly more than other factors such as hypertension, smoking, or previous cardiovascular diseases. Some authors also mentioned that pulmonary function decline is associated with higher risk for renal failure (43), diabetes (44), and neurocognitive diseases (45). In light of the abovementioned and our findings, it seems probable to conclude that higher work-related PA results in better health during the retirement among blue-collar workers.

Our study is not without limitations. First of all, the “healthy user bias” might have occurred due to the restrictive selection criteria of the retirees (exclusion criteria). Individuals with respiratory system diseases were excluded to avoid bias in spirometric measurements. In consequence, the influence of occupation type on developing such diseases cannot be assessed. Next, as the group of retirees participating in the study was relatively young, it is possible that the results of this study may not reflect the condition of oldest-old retired people. Another limitation is that, lifetime physical activity was evaluated in a subjective manner. Despite having made every effort to help the subjects recall as many details as possible and having conducted a detailed interview, the activities may have been inaccurately reported by the respondents. Regardless, due to the nature of this cross - sectional study assessing past physical activity, it was not possible to apply more objective tools to evaluate the activity or physical exertion of the subjects. Smoking was not analyzed in two occupational groups in this study but was one of the exclusion criteria., the data on the distribution of smokers among occupational groups were not available. Other studies (46–48) indicates that blue - collar workers presented higher smoking prevalence than white - collar workers. As indicated by de Castro et al. (46) blue - collar work may increase the risk of being a smoker up to 2.5 times, so it can also be assumed that it may increase the risk of smoking-related respiratory diseases, which may reduce the physical fitness of the older adults. However, due to lack of the data, in present study it is not possible to assess the abovementioned relationships of smoking.

In conclusion, in our study, we found no statistically significant relationship between the retired white- and blue- collar workers in terms of physical performance scores. No differences in the HGS, SPPB, FEV, FEV*1, and IVC scores were found, despite statistically significant differences between mean intensity (MET/week/year) and duration (h/week/year) of sports- and work-related lifetime physical activity. Only association between type of occupation and membership in SPPB score quartiles in favor of white – collar workers was found. The results of present study are important for healthcare practitioners and workers, and are indicating that both blue- and white- collar workers should maintain high non-work related PA, despite different occupational PA levels. These findings may indicate that the type of past work is not an independent factor influencing the state of a person in old age. Blue-collar workers who had worked with higher intensity presented higher FEV*1 scores after retiring, while white-collar workers who had spent less time at work presented higher HGS after retiring.

As the number of the literature reports about the effects of occupational activity on the health of older people after retiring is limited, it seems prudent to conduct further, large-scale investigations in large sample-size groups, with physically fit and unfit subjects, not just healthy individuals.

## Data Availability Statement

The raw data supporting the conclusions of this article will be made available by the authors, without undue reservation.

## Ethics Statement

The studies involving human participants were reviewed and approved by Bioethics Committee at Poznan University of Medical Sciences. The patients/participants provided their written informed consent to participate in this study.

## Author Contributions

TT, EZ, and MP developed a study design. TT and AP performed measurements and data collection. AP performed statistical analysis of the data. TT was a major contributor in writing the manuscript. All authors performed interpretation of the data, read and approved the final manuscript.

## Funding

This work was supported by the Poznan University of Medical Science under the Young Scientists (Grant No. 502-14-44275460-11095).

## Conflict of Interest

The authors declare that the research was conducted in the absence of any commercial or financial relationships that could be construed as a potential conflict of interest.

## Publisher's Note

All claims expressed in this article are solely those of the authors and do not necessarily represent those of their affiliated organizations, or those of the publisher, the editors and the reviewers. Any product that may be evaluated in this article, or claim that may be made by its manufacturer, is not guaranteed or endorsed by the publisher.

## Abbreviations

EU: European Union
PA: Physical Activity
WHO: World Health Organization
IPAQ: International Physical Activity Questionnaire
COPD: Chronic Obstructive Pulmonary Disease
LTPAQ: Lifetime Physical Activity Questionnaire
MET: Metabolic Equivalent of Task
ISCO-08: The International Standardized Classification of Occupations
SPPB: Short Physical Performance Battery test
HGS: Hand-Grip Strength
ASHT: American Society of Hand Therapists
FVC: Forced Vital Capacity
FEV*1: Forced Expiratory Volume in the 1st second
IVC: Inspiratory Vital Capacity
GLR: General Linear Regression.
